# Semi-Supervised Maximum Discriminative Local Margin for Gene Selection

**DOI:** 10.1038/s41598-018-26806-6

**Published:** 2018-06-05

**Authors:** Zejun Li, Bo Liao, Lijun Cai, Min Chen, Wenhua Liu

**Affiliations:** 1 College of Information Science and Engineering, Hunan University, Changsha, Hunan 410082 China; 2 School of Computer and Information Science, Hunan Institute of Technology, Hengyang, 412002 China

## Abstract

In the present study, we introduce a novel semi-supervised method called the semi-supervised maximum discriminative local margin (semiMM) for gene selection in expression data. The semiMM is a “filter” approach that exploits local structure, variance, and mutual information. We first constructed a local nearest neighbour graph and divided this information into within-class and between-class local nearest neighbour graphs by weighing the edge between the two data points. The semiMM aims to discover the most discriminative features for classification via maximizing the local margin between the within-class and between-class data, the variance of all data, and the mutual information of features with class labels. Experiments on five publicly available gene expression datasets revealed the effectiveness of the proposed method compared to three state-of-the-art feature selection algorithms.

## Introduction

Currently, the expression level of hundreds of thousands of genes can be successfully monitored with popular high-throughput technology in a single experiment. This technology is widely used in the post-genomic era for related disease research^1–3^. Only a few genes can cause disease^4^. The gene expression levels of these disease-causing genes greatly vary between positive and negative samples^5,6^. Therefore, the classification of tumour tissue or other diseases by analysing differential expression data and identifying disease-causing genes is attractive and practically meaningful^7–9^. Curse-of-dimensionality may occur during the classification phase due to a property of gene expression data that states that a small sample size has high dimensionality^10^. Various dimension-reduction methods have been developed to avoid this phenomenon.

Feature selection is a dimension-reduction technique that evaluates features using proper optimization criteria^11,12^, such as variance criteria^13^, maximum local margin criteria, mutual information criteria^14–16^, and correlation criteria^17^. Feature selection methods contain wrapper^18^ and filter methods^19–21^. Compared to wrapper methods, filter methods are efficient and simple due to their classifier-independent feature selection. In addition, manually labelling a positive or negative sample is both time- and labour-consuming. Thus, gene expression data lack labelled samples but have abundant unlabelled samples. Current studies have attempt to uncover the most discriminative information from all samples. Although some supervised and unsupervised feature selection methods can perform well, utilizing the information of both labelled and unlabelled data can enhance their performance^22–24^. This point was verified in a series of different environmental settings of the Fisher criterion^25–28^. These three studies showed that the local manifold structure is useful for selecting more informative genes, and the discriminative power can be increased in a local semi-supervised manner.

Motivated by the maximum margin projection (MMP)^29^, Laplacian score (LS)^30^, and mutual information technique, we proposed a novel semi-supervised gene selection method called the semi-supervised maximum discriminative local margin (semiMM). The semiMM can be used for tumour classification or analysis of differential gene expression levels. This method aims to maximize the local margin between within-class and between-class data and simultaneously discover the most closely related class features. The features were evaluated according to their contribution to the local margin that preserves power and class discriminative capability. Specifically, the maximum local margin is designed to maintain the consistency in local geometrical structure of the same class and the separability of different classes. In maximizing the mutual information between classes and features, the relationship between class labels and features is considered to achieve increased discriminative power.

The present review is structured as follows. Work-related dimensionality reduction methods are briefly reviewed in Section 2. The proposed semiMM algorithm is introduced in Section 3. Experiments on five publicly available gene expression datasets are presented in Section 4. Finally, the conclusions from the present study and suggestions for future work are discussed in Section 5.

## Related Studies

In this section, we present a brief review of two dimensionality reduction methods, namely, the LS^30^ and MMP^29^, which are related to the proposed semiMM.

### Notations

In the present study, matrix ${\rm{X}}=\{{{\rm{x}}}_{1},\,{{\rm{x}}}_{2},\cdots ,\,{{\rm{x}}}_{{\rm{m}}}\}\,\in \,{{\rm{R}}}^{{\rm{n}}\times {\rm{m}}}$ refers to the gene expression data, where *m* denotes the number of samples, and *n* denotes the number of genes, which is the dimensionality number. ${{\rm{f}}}_{r}={[{f}_{r1},{f}_{r2},\cdots ,{f}_{rm}]}^{T}\,\in \,{{\rm{R}}}^{n}$ is an *n* dimensional column vector that denotes the *rth* gene in the gene expression data, where *f*_*ri*_ indicates the *rth* gene in the *ith* sample. The matrix is presented by boldface and capital letters, whereas the vectors are denoted by boldface and lowercase letters.

### Maximum Margin Projection

The MMP is a semi-supervised learning method for dimensionality reduction. This semi-supervised learning method has two assumptions: smoothness and cluster^31^. The former indicates that if two points are close to each other in a high-density region, then the corresponding projecting outputs should also be close. The latter assumes that the points in the same cluster tend to be in the same class. MMP obeys these two rules and aims to capture both the geometrical and discriminating structures of the local data manifold with both labelled and unlabelled data.

The MMP constructs a k nearest neighbour graph *G* with a binary weight to depict the geometry of the underlying local manifold. *G* is divided into two subgraphs, that is, the within-class graph *G*_*w*_ and between-class graph *G*_*b*_, to discover the discriminating information of the data manifold. *N*(*x*_*i*_) denotes the k nearest neighbours of arbitrary data point *x*_*i*_ and is naturally composed of *N*_*b*_(*x*_*i*_) and *N*_*w*_(*x*_*i*_). If the samples are neighbours and have different class labels, then they belong to set *N*_*b*_(*x*_*i*_); otherwise, the remaining neighbours are placed into *N*_*w*_(*x*_*i*_). *W*_*b*_ and *W*_*w*_ are the weight matrices of *G*_*b*_ and *G*_*w*_, respectively, with the following definitions:

1 $${W}_{b,ij}=\{\begin{array}{c}1,\,\,if\,{x}_{i}\in {N}_{b}({x}_{j})\,or\,{x}_{j}\in {N}_{b}({x}_{j}),\\ 0,\,\,otherwise.\,\end{array}$$

2 $${W}_{w,ij}\{\begin{array}{c}\gamma ,\,\,if\,{x}_{i}\,and\,{x}_{j}\,share\,the\,same\,class\,label,\\ 1,\,\,if\,{x}_{i}\,or\,{x}_{j}\,is\,unlabeled\,but\,{x}_{i}\in {N}_{w}({x}_{j})\,or\,{x}_{j}\in {N}_{w}({x}_{i}),\\ 0,\,\,otherwise.\end{array}$$

Semi-supervised graph embedding is similar to locality sensitive discriminant analysis (LSDF), a semi-supervised feature selection algorithm proposed in^32^.

MMP detects a linear transformation based on the following two objective functions to maximize the local margin between the within-class graph *G*_*w*_ and between-class graph *G*_*b*_:

3 $${\rm{\min }}\,\frac{1}{2}{\sum }_{ij}{({a}^{T}{x}_{i}-{a}^{T}{x}_{j})}^{2}{W}_{w,ij}$$

4 $${\rm{\max }}\,\frac{1}{2}{\sum }_{ij}{({a}^{T}{x}_{i}-{a}^{T}{x}_{j})}^{2}{W}_{b,ij}$$

where *a* is a projection vector of projection matrix A and A ∈ R^*d*×*n*^. By performing some algebraic steps and imposing a constraint, *a*^*T*^*XD*_*w*_*X*^*T*^*a* = 1, the objective functions (3) and (4) can be rewritten as (5) and (7), respectively:

5 $${\min }_{{\rm{a}}}1-{a}^{T}X{W}_{w}{X}^{T}a$$

Equivalent to

6 $${\max }_{{\rm{a}}}{a}^{T}X{W}_{w}{X}^{T}a$$

7 $${\max }_{{\rm{a}}}{a}^{T}X{W}_{b}{X}^{T}a$$

Thus, the optimization problem is:

8 $$\text{arg}\,{\max }_{{a}^{T}X{D}_{W}{X}^{T}a=1}\,{a}^{T}X(\alpha {W}_{b}+(1-\alpha ){W}_{w}){X}^{T}a$$

9 $$X(\alpha {W}_{b}+(1-\alpha ){W}_{w}){X}^{T}a=\gamma X{D}_{w}{X}^{T}a$$

where α is a tuning constant with 0 ≤ α ≤ 1. The optimal projection vector a is subsequently obtained by solving the generalized eigenvalue problem defined in Eq. (9), where *γ* is the generalized eigenvalue. This linear transformation can optimally and simultaneously preserve the local neighbourhood and discriminatory information.

### Laplacian Score

The LS is an unsupervised feature selection method proposed in^30^. This method was developed due to the observation that two data points close to each other are potentially in the same class. The LS selects features with more locality preserving power as evaluated by Eq. (10). Moreover, the LS is similar to two pop manifold learning methods, namely, Laplacian eigenmaps^33^ and locality preserving projection^34^. The LS first constructs a k nearest neighbour graph, which is defined in Eq. (11). Given that the variance in the data manifold can be calculated by Eq. (12) based on the spectral graph theory^35^, Eq. (10) can be reformulated as Eq. (13) by performing some algebraic steps.

10 $${L}_{r}=\frac{{\sum }_{ij}{({f}_{ri}-{f}_{rj})}^{2}{W}_{ij}}{Var({f}_{r})}$$

11 $${W}_{ij}=\{\begin{array}{l}{e}^{-\frac{{({x}_{i}-{x}_{j})}^{2}}{t},if{x}_{j}\in {N}_{k}({x}_{i})or{x}_{i}\in {N}_{k}({x}_{j})},\\ 0,\,otherwise.\end{array}$$

12 $${\rm{Var}}({f}_{r})={\sum }_{i}{({f}_{ri}-{\mu }_{r})}^{2}{D}_{ii}={\sum }_{i}{f}_{ri}^{ \% 2}{D}_{ii}$$

13 $${L}_{r}=\frac{{f}_{r}^{ \% T}L{f}_{r}^{ \% }}{{f}_{r}^{ \% T}D{f}_{r}^{ \% }}$$

where *D* is a diagonal matrix with ${D}_{ii}={\sum }_{j}{W}_{ij}$, and *L* is a Laplacian matrix with a definition of *L* = *D* − *W*.

Specifically, a “good” feature indicates more representative power and local structure preserving power. The former requires larger variance of a feature, and the latter means that if two data points are very close, then these points should have similar features. In an algebraic sense, increased representative power and local structure preserving power can be interpreted as maximizing the denominator and minimizing the numerator in Eq. (10). Consequently, feature selection with the LS is performed to minimize the objective function in Eq. (10); that is, a smaller *L*_*r*_ indicates that better features are selected.

## Semi-Supervised Maximum Discriminative Information for Feature Selection

In this section, we introduce the proposed semiMM from two aspects, including the criterion and algorithm flow of the semiMM.

The semiMM is a semi-supervised feature selection method based on manifold learning. The graph embedding originated from the previously described MMP, which is a semi-supervised manifold learning method (see Section 2). Thus, the semiMM constructs between-class and within-class neighbour graphs to simultaneously characterize the local manifold of the dataset with all samples and the discriminative information from the labelled samples. Moreover, the semiMM also considers the variance of features and the mutual information between the classes and features. This method aims to maximize the local margin between within-class and between-class data and simultaneously discover the most related class features.

### Criterion of SemiMM

Based on the two basic assumptions about semi-supervised learning mentioned in Section 2, two data points from the same neighbourhood potentially belong to the same class (and vice versa) with the name of the local preserving power. A “good” feature possesses more local preserving power and is most discriminative in clarifying the data.

Therefore, the within-class and between-class information should be simultaneously minimized and maximized, respectively, to ensure a maximum local margin. In addition, a good feature for gene selection should be genes differentially expressed for samples with different class labels. This difference in gene expression level can be characterized by the mutual information between features and class labels, denoted by *NMI*(*f*_*r*_, c). A larger difference indicates more mutual information and vice versa. Maximizing the mutual information between features and class labels might enhance the discriminative capability. A reasonable criterion of the semiMM is to minimize the objective function given as follows:

14 $$semiM{M}_{r}=\lambda \frac{{\sum }_{i,j=1}^{m}{({f}_{ri}-{f}_{rj})}^{2}{W}_{w,ij}-{\sum }_{i,j=1}^{m}{({f}_{ri}-{f}_{rj})}^{2}{W}_{b,ij}}{Var({f}_{r})}+(1-{\rm{\lambda }})(1-NMI({f}_{r}\,,\,C)$$

The first term in Eq. (14) shares the same idea with the LS, which regards variance information as a representative power of all data points. The first term in our objective function represents the local margin preserving power. The second term characterizes the class-related capability, where λ is a tuning parameter with 0 < λ < 1, and *semiMM*_*r*_ denotes the score of the λth feature evaluated by the proposed semiMM.

Given *S* = *W*_*w*_ − *W*_*b*_, the objective function can be rewritten as Eq. (15) through some simple algebraic steps, where L is the Laplacian matrix with *L* − *D* − *S*, and D is a diagonal matrix with the column or row sum of the symmetric matrix *S*_*ij*_ being its diagonal entries. The normalized mutual information between features and class labels can be calculated by Eq. (16):

15 $${{\rm{semiMM}}}_{{\rm{r}}}={\rm{\lambda }}\frac{{{\rm{f}}}_{{\rm{r}}}^{{\rm{T}}}{{\rm{Lf}}}_{{\rm{r}}}}{{{\rm{f}}}_{{\rm{r}}}^{ \% {\rm{T}}}{{\rm{Df}}}_{\,r}^{ \% }}+(1-{\rm{\lambda }})(1-{\rm{NMI}}({{\rm{f}}}_{{\rm{r}}},\,{\rm{C}}))$$

16 $${\rm{NMI}}({{\rm{f}}}_{{\rm{r}}},\,{\rm{C}})=\frac{{\rm{MI}}({{\rm{f}}}_{{\rm{r}}},\,{\rm{C}})}{{\rm{\max }}({\rm{H}}({{\rm{f}}}_{{\rm{r}}}),\,{\rm{H}}({\rm{C}}))}$$

17 $${{\rm{f}}}_{{\rm{r}}}^{ \% }={{\rm{f}}}_{{\rm{r}}}-{{\rm{\mu }}}_{{\rm{r}}}1={{\rm{f}}}_{{\rm{r}}}-\frac{{{\rm{f}}}_{{\rm{r}}}^{{\rm{T}}}{\rm{D}}1}{{1}^{{\rm{T}}}{\rm{D}}1}$$

### Algorithm flow of SemiMM

In summary, the algorithm flow of the semiMM is presented as follows:

**Algorithm 1:**
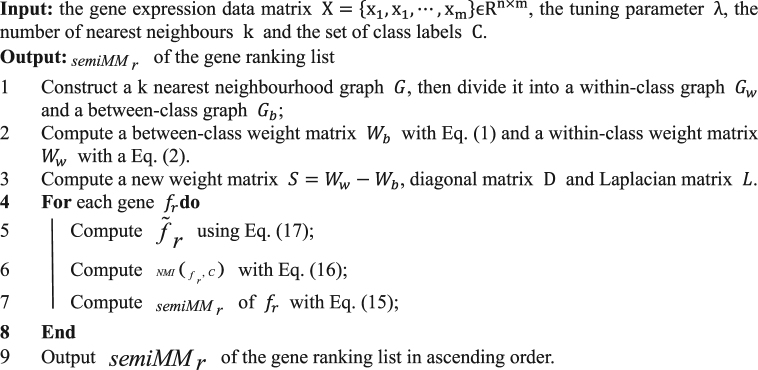
Semi-Supervised Maximum Discriminative Local Margin Feature Selection Algorithm.

## Experiments

In this section, we conducted extensive experiments to evaluate the performance of the proposed semiMM method in a semi-supervised manner for gene selection. Experiments are conducted on five gene expression profile datasets. All datasets are publicly available from GEMS^36^. The detailed description of the datasets is shown in Table 1.

**Table 1:** Dataset descriptions, including the sample number, gene dimension and class number.

DataSet	Num of Sample	Num of Dim	Num of Class
DLBCL	77	5469	2
Prostate_Tumor	102	10509	2
Leukemia2	72	11225	3
SRBCT	83	2308	4
Lung_Cancer	203	12600	5

The methods presented in this article were evaluated using five tumour datasets and compared to other methods. The following is a brief introduction to the five datasets used in this article.

1. DLBCL: This dataset is a two-class dataset with two subclasses DLBCL (0) and FL (1) (the numbers in parentheses indicate the class labels in all datasets). The dataset contains a total of 77 samples, the DLBCL and FL sample ratio is 58:19, and the total number of genes is 5469.
2. Prostate_Tumor: This dataset is also a two-class dataset; the two sub-categories are tumour samples, Tumour (0), and normal samples, Normal (1). The dataset contains a total of 102 samples, the Tumour and Normal sample ratio is 50:52, and the total number of genes is 10,509.
3. Leukemia2: This dataset is a three-class dataset, and the three subclasses are AML (0), ALL (1) and MLL (2). The dataset contains 72 samples, and the total number of genes is 11,225.
4. SRBCT: This dataset is a four-class dataset, and the four subclasses are EWS (0), RMS (1), BL (2), and NB (3). The dataset contains a total of 83 samples, including EWS, RMS, BL and NB at a sample ratio of 29:25:11:18, and the total number of genes is 2308.
5. Lung_Cancer: This dataset is a five-class dataset, with four subclasses, including Adeno (0), Normal (1), Squamous (2), COID (3) and SMCL (4). This dataset contains 203 samples, and the total number of genes is 12,600.

### Experimental Design

In this experiment, we first pre-processed the five gene expression datasets to obtain the prepared data: initial data for feature selection and split data for classification. The optimal values of parameters were selected in the proposed method. Three state-of-the-art feature selection methods were selected for comparison to better understand the proposed method. The experiments were conducted, and the outputs were recorded and analysed.

### Data Preparation

#### Initial Data

In this experiment, we set up a semi-supervised setting to simulate the “small sample, high dimension” problem. In a semi-supervised setting for a filtered feature selection, both labelled and unlabelled samples must be used during the calculation of the score of each feature, and the feature selection method is used to rank the features. Here, we selected different numbers of samples per subclass of a gene expression dataset, in which the labelled data with stratified random sampling is denoted by L. The values of L are 2, 4, and 6. Thus, the number of labelled samples in a certain gene expression dataset is the product of L and the number of classes. The remaining data in the dataset are regarded as unlabelled data. The obtained data were termed initial data for convenience.

#### Split Data

During classification, we divided each gene expression dataset into a training and testing set with a ratio of 6:4 through stratified random sampling. We conducted the classification with different numbers of genes ranging from 5 to 300 with a step of 5. Considering the intrinsic characteristics of semi-supervised feature selection, we repeated the experiment 10 times at each step and recorded the average prediction accuracy for evaluation.

### Compared Methods and Experimental Setup

#### Laplacian score

The LS is an unsupervised feature selection method. In this method, a nearest neighbour graph is constructed to model the local geometric structure^30,37^. The LS selects the features with more locality preserving power^25^.

#### Fisher score

As a supervised feature selection method, the Fisher score seeks features according to their discriminating power^32^.

#### LSDF

As a semi-supervised feature selection algorithm, the LSDF utilizes both labelled and unlabelled data and determines the discriminative structure and geometrical structure of the data. Features that can maximize the margin between the within-class and between-class graphs are selected by the LSDF.

In the proposed semiMM, the tuning parameter lambda can be searched from the grid {0.1, 0.2, 0.3, 0.4, 0.5, 0.6, 0.7, 0.8, 0.9}. The number of nearest neighbours, k, is empirically assumed to be 5 because the k nearest neighbours are adopted to model the local manifold structure of the data. The weight in the whole experiment is determined by binary similarity, and the alpha is set as 100, similar to LSDF. By conducting many experiments to select the proper value of parameters in the proposed algorithm, we determined that the proposed method can robustly detect changes of the parameters, whereas the LSDF is sensitive to k and alpha. Thus, k and alpha are set at 5 and 100, respectively, to ensure that the LSDF can still perform well in the experiments, and a better comparison between LSDF and the proposed semiMM can be obtained. In this experiment, the top 300 genes were selected as the feature subset for classification, and each gene was normalized to achieve zero mean and unit variance for further assessment.

### Evaluation Metrics

In this evaluation framework, five evaluation metrics, including accuracy, precision, recall, f-score, and area under the receiver operating characteristic curve (AUC), were used to assess the performance. These metrics were determined by the following equations:

18 $$accuracy=\frac{TP+TN}{TP+TN+FN+FP}$$

19 $$precision=\frac{TP}{TP+FP}$$

20 $$recall=\frac{TP}{TP+FN}$$

21 $$f-score=\frac{2\,\ast \,precision\,\ast \,recall}{precision+recall}$$

where true positives and true negatives refer to the number of samples that are correctly classified into their class group in the ground truth; i.e., positive samples are predicted to be positive, and negative samples are classified into the negative group. The same logic is applied to understand false negatives (FN) and false positives (FP). The tumour dataset Lung is an unbalanced multiclass dataset. As stated in^32^, a larger AUC indicates better performance. Thus, the AUC score is applied to assess the prediction performance of classification to properly evaluate FPs and FNs for cancer classification.

The proposed semiMM can manage both binary classification and multi-classification datasets. The gene expression datasets used in the present study include two binary classification datasets and three multi-classification datasets. To perform the multi-classification experiment, we devised a one-against-rest approach for each class and thus constructed c binary classifiers, where c denotes the number of classes in each dataset. The average results over the c binary classifiers are shown as the final result of multi-classification.

### Experimental Results

**Figure 1:**
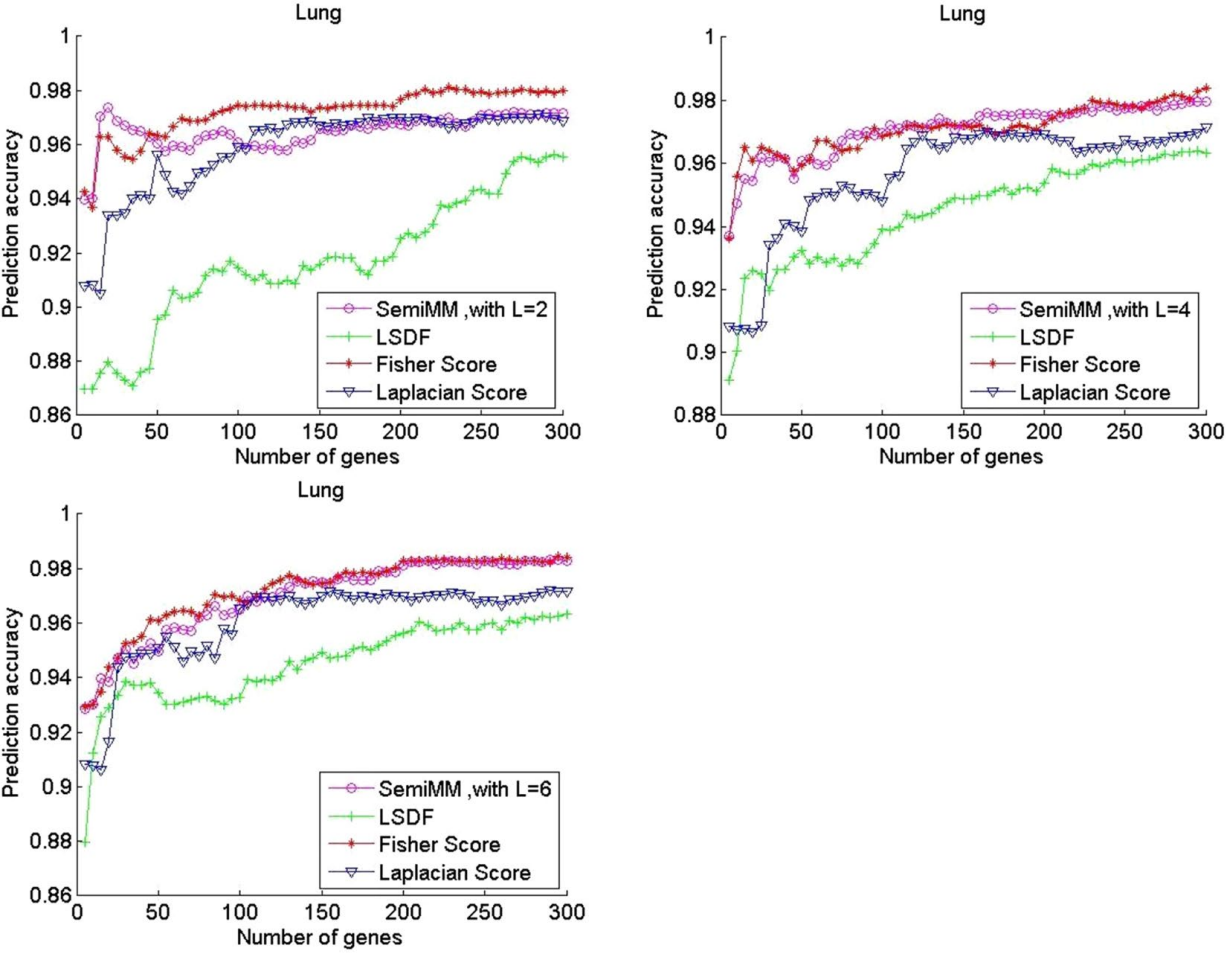
Performance comparison of average prediction accuracy of binary classification gene expression datasets Prostate.

In this subsection, classification is performed via SVM on the training set with a chosen feature subset (the top 300 genes) in the five gene expression datasets to evaluate the performance of the proposed semiMM method and compare it with three other methods. Figure 1 shows the curves of average prediction accuracy versus gene dimension for the four methods with different labelled samples on two binary classification datasets.

**Figure 2:**
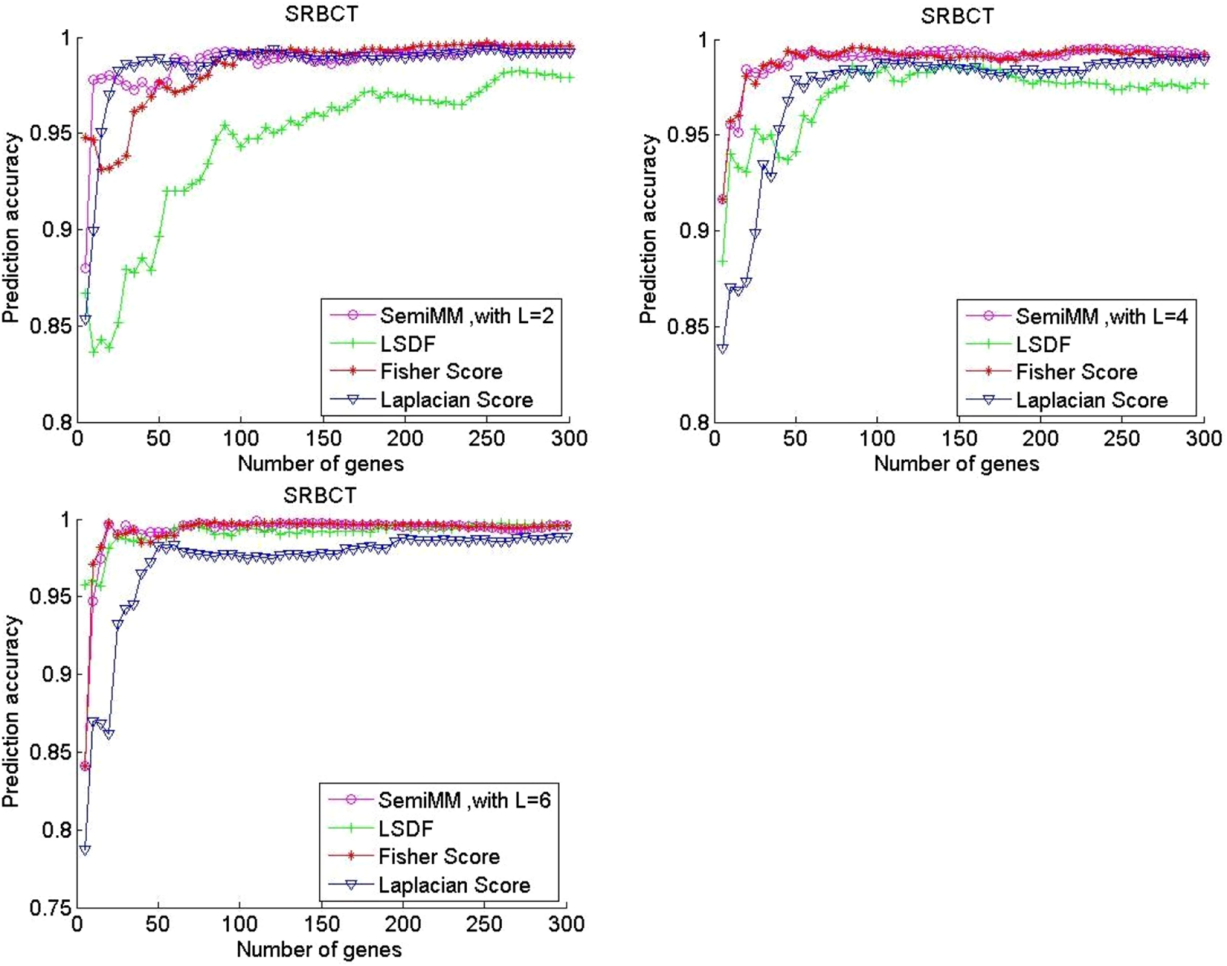
Performance comparison of average prediction accuracy of binary classification gene expression datasets DLBCL.

All filter methods achieve high average prediction accuracy with an increased number of selected genes in most cases. Figures 1 and 2 shows that the performance of the supervised Fisher score method is improved when the number of labelled samples per subclass L increases from 2 to 6. In contrast, the performance of the unsupervised LS method has degraded. A larger L value indicates that fewer unlabelled samples remained in each dataset. Thus, the observation is reasonable. However, the semiMM and LSDF methods perform better with a larger L.

The semiMM method performs best and converges fastest to the optimal point when less than 100 genes are selected. This finding might indicate that the proposed semiMM method has better ability to utilize the label information than LSDF, i.e., the semiMM has more discriminating power than the LSDF method. Roughly speaking, the semiMM and LSDF show stable performance with varying values of L because the shapes of their curves are almost unchanged. This finding can be explained by the semi-supervised properties of the semiMM and LSDF; these methods simultaneously select features from both labelled and unlabelled samples.

**Figure 3:**
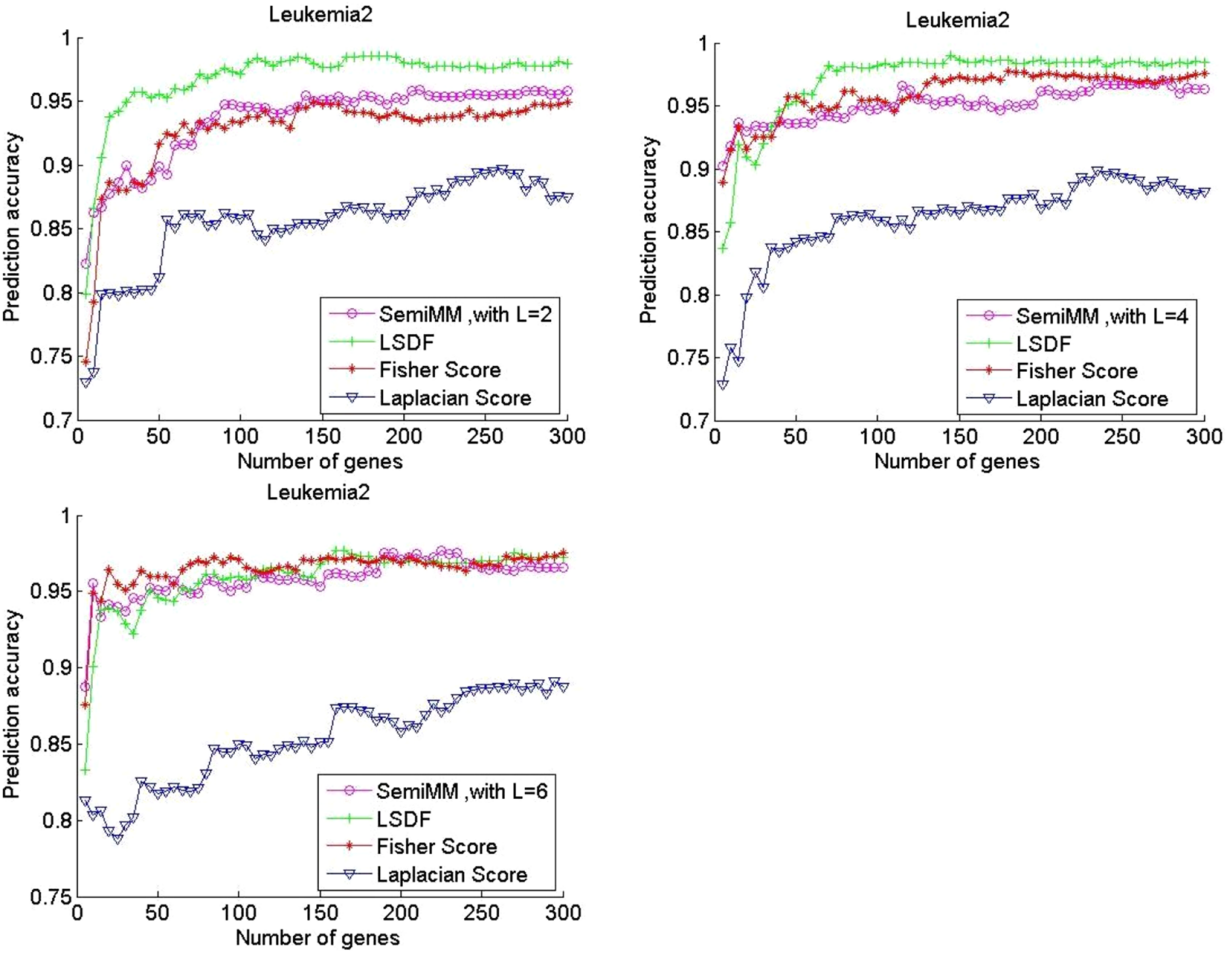
Performance comparison of average prediction accuracy in multi-classification gene expression datasets Leukemia2.

**Figure 4:**
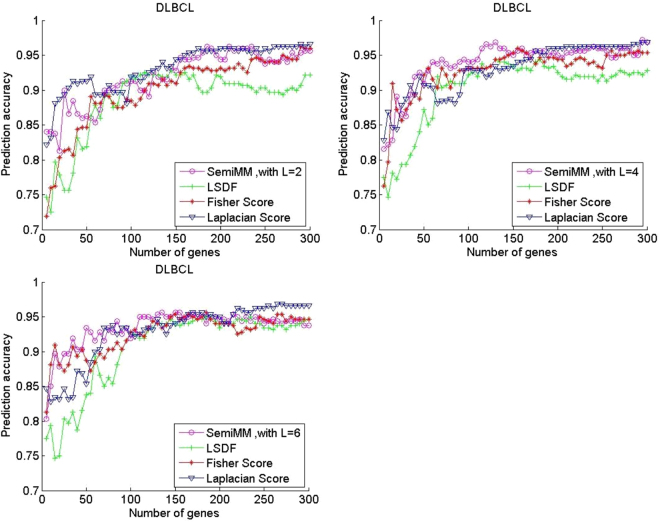
Performance comparison of average prediction accuracy in multi-classification gene expression datasets SRBCT.

**Figure 5:**
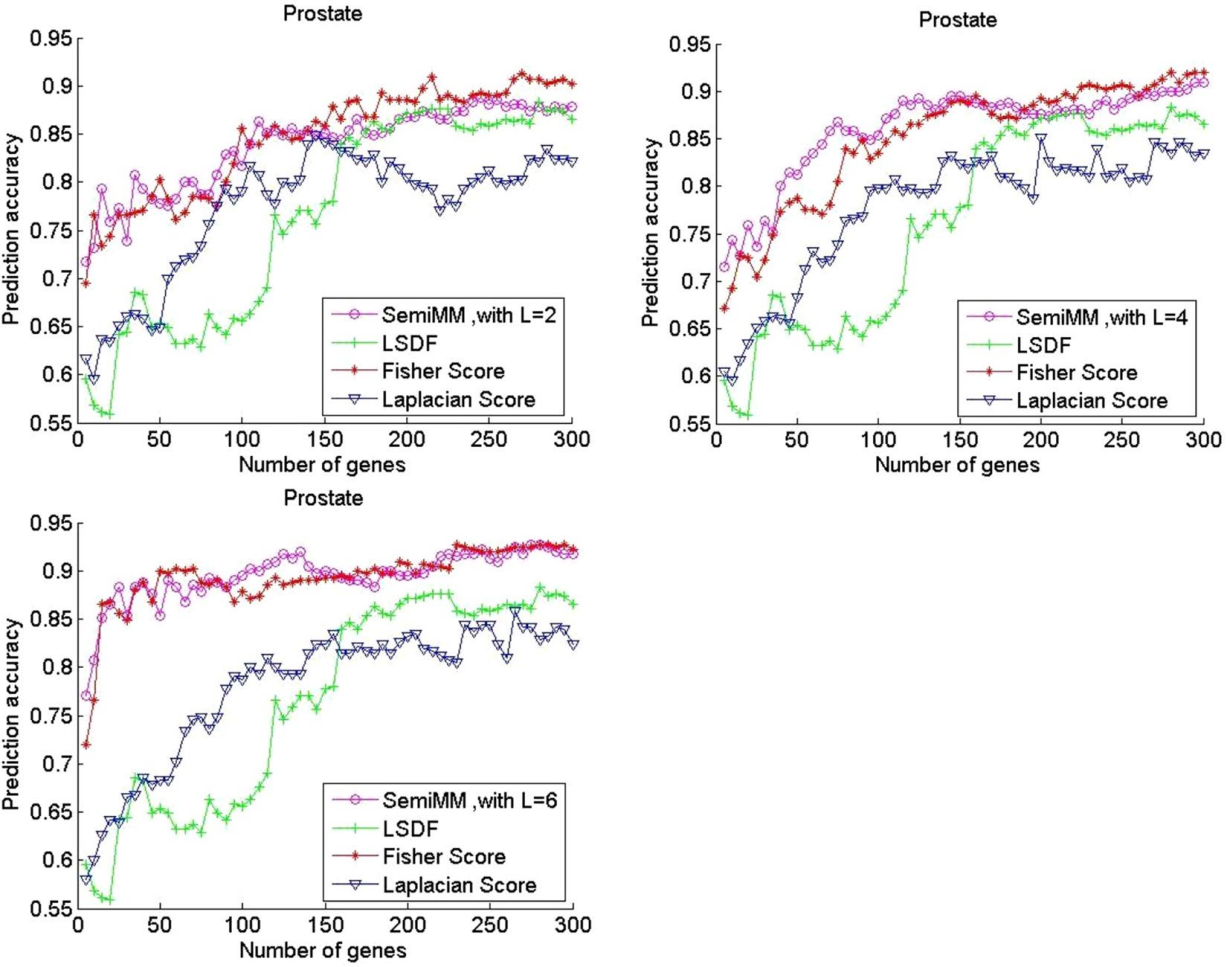
Performance comparison of average prediction accuracy in multi-classification gene expression datasets Lung.

The multiclass classification performance of three publicly available datasets is shown in Figs 3–5. The performance of the LS is unchanged in all three multiclass datasets, and its average prediction accuracy decreases when additional labelled samples are selected. The Fisher score performance improves with a larger L on the Leukemia2 and SRBCT datasets but degrades slightly on the Lung dataset under the same condition. Thus, not all labelled samples are useful for category recognition. Overall, the proposed semiMM method converges faster and achieves slightly higher optimal average classification accuracy when L increases in all three multiclass datasets. When L equals 2, the semiMM outperforms the supervised Fisher score method when the number of selected genes is less than 50 for multiclass datasets. The performance of the other semi-supervised method, LSDF, is slightly different; its average classification accuracy is poor when L increases in the Leukemia2 dataset but is totally different on the SRBCT and Lung datasets. In addition, its performance is not comparable to that of the other methods in most cases.

Therefore, the semiMM performs well irrespective of the dataset itself, whereas its competitors are sensitive to the dataset. The semiMM is effective for tackling “small sample” problems. The good and stable performance of this method is due to its simple and efficient idea to discover both geometrical and discriminating information with labelled and unlabelled samples together. Although no method outperforms the other three algorithms in all circumstances, with regard to robustness of the dataset and good prediction accuracy, and the proposed semiMM is a good choice for gene selection with small and limited numbers of labelled samples.

Considering that all four methods show a stable and promising performance when the number of selected genes is 150, we listed the corresponding classification results with different values of L in Tables 2–6. For a given L, the highest values are shown in bold-faced forms. The parameter λ is set as 0.6 in the proposed semiMM in all experiments. From the binary datasets, i.e., Tables 2 and 3, and the following three multiclass datasets, the semiMM and Fisher score achieve the highest values in most cases. In the cases where the semiMM is not the best method, its performance remains higher and better than that of the other two. This finding verifies the conclusion from the analysis of Figs 1 and 2. The proposed semiMM is an effective feature selection method with good and stable performance irrespective of the dataset itself.

**Table 2:** Comparison of mean evaluation metrics of the DLBCL dataset with the top 150 selected genes by varying the value of L.

	Acc labelNum = 2/4/6	Precision labelNum = 2/4/6	Recall labelNum = 2/4/6	F-score labelNum = 2/4/6	AUC labelNum = 2/4/6
semiMM	0.9281**/0.9500/0.9563**	0.8269/0.8989/0.8865	**0.9125**/0.9125/**0.9500**	0.8630/**0.9009/0.9144**	0.9818/0.9844/0.9833
LSDF	0.9219/0.9344/0.9406	0.8436/0.8903/0.8856	0.8625/0.8500/0.8875	0.8487/0.8634/0.8797	0.9568/0.9802/0.9813
Fishser	0.9094/**0.9531**/0.9531	0.8340/**0.9182/0.8944**	0.8250/0.9000/0.9250	0.8193/**0.9069**/0.9080	0.9573/**0.9859**/0.9823
Laplacian	**0.9438**/0.9344/0.9406	**0.8791**/0.8360/0.8672	**0.9125/0.9250**/0.9125	**0.8893**/0.8752/0.8865	**0.9865**/0.9828/**0.9854**

**Table 3:** Comparison of the mean evaluation metrics of the Prostate dataset with the top 150 selected genes by varying the value of L.

	Acc labelNum = 2/4/6	Precision labelNum = 2/4/6	Recall labelNum = 2/4/6	F-score labelNum = 2/4/6	AUC labelNum = 2/4/6
semiMM	0.8512**/0.8951/0.9000**	0.8394/0.8849**/**0.8972	0.8600**/0.9050/0.9000**	0.8486/**0.8941/0.8980**	0.9052**/0.9390/0.9531**
LSDF	0.7780/0.7780**/**0.7780	0.7671/0.7671**/**0.7671	0.8000/0.8000**/**0.8000	0.7799/0.7799**/**0.7799	0.8407/0.8407**/**0.8407
Fishser	**0.8585**/0.8902**/**0.8927	**0.8466/0.9083/0.9010**	**0.8700**/0.8650**/**0.8800	**0.8571**/0.8853**/**0.8888	**0.9219**/0.9188**/**0.9474
Laplacian	0.8415/0.8244**/**0.8244	0.8426/0.8313**/**0.8277	0.8300/0.8050**/**0.8100	0.8358/0.8155**/**0.8180	0.8926/0.8981**/**0.8850

**Table 4:** Comparison of mean evaluation metrics in the Leukemia2 dataset with the top 150 selected genes by varying the value of L.

	Acc labelNum = 2/4/6	Precision labelNum = 2/4/6	Recall labelNum = 2/4/6	F-score labelNum = 2/4/6	AUC labelNum = 2/4/6
semiMM	0.9511/0.9556**/**0.9533	0.94670.9440**/**0.9369	0.9028/0.9169**/**0.9103	0.9201/0.9271**/**0.9198	0.9774/0.9885**/**0.9867
LSDF	**0.9767/0.9867/**0.9678,	**0.9718/0.9875/0.9701**	**0.9625/0.9694/**0.9278	**0.9653/0.9765/**0.9454	0.9974/0.9989**/**0.9938
Fishser	0.9478/0.9733**/0.9711**	0.9514/0.9670**/**0.9646	0.8894/0.9414**/0.9392**	0.9164/0.9527**/0.9498**	0.9795/0.9939**/**0.9895
Laplacian	0.8533/0.8644**/**0.8511	0.7544/0.7731**/**0.7492	0.7728/0.7950**/**0.7789	0.7570/0.7760**/**0.7605	0.9125/0.9159**/**0.9003

**Table 5:** Comparison of mean evaluation metrics of the SRBCT dataset with the top 15 selected genes by varying the value of L.

	Acc labelNum = 2/4/6	Precision labelNum = 2/4/6	Recall labelNum = 2/4/6	F-score labelNum = 2/4/6	AUC labelNum = 2/4/6
semiMM	0.9879**/0.9943/0.9971**	0.9939**/1/1**	0.9654/**0.9808/0.9917**	0.9785**/0.9896/0.9953**	0.9995/**1**/0.9998
LSDF	0.9593/0.9850/0.9921	0.9657/0.9864/0.9914	0.8854/0.9654/0.9833	0.9208/0.9746/0.9865	0.9863/0.9980/0.9998
Fishser	**0.9921**/0.9907/0.9964	**1**/0.9975/**1**	**0.9708**/0.9713/0.9896	**0.9843**/0.9834/0.9943	**1**/0.9997**/1**
Laplacian	0.9886/0.9850/0.9786	0.9942/0.9952/0.9816	0.9706/0.9556/0.9435	0.9808/0.9736/0.9600	0.9993/0.9978/0.9971

**Table 6:** Comparison of mean evaluation metrics of the Lung dataset with the top 150 selected genes by varying the value of L.

	Acc labelNum = 2/4/6	Precision labelNum = 2/4/6	Recall labelNum = 2/4/6	F-score labelNum = 2/4/6	AUC labelNum = 2/4/6
semiMM	0.9660**/0.9723/0.9749**	0.9326**/0.9552/0.9606**	0.8561**/0.9077/0.9300**	0.8811**/0.9247/0.9415**	0.9878/0.9898/0.9898
LSDF	0.9157**/**0.9487**/**0.9494	0.6887**/**0.9228**/**0.9254	0.5888/0.8027/0.8044	0.6221/0.8463/0.8454	0.8898/0.9607/0.9623
Fishser	**0.9737/**0.9720**/**0.9747	**0.9445/**0.9550**/**0.9602	**0.8794**/0.9055/0.9237	**0.8985**/0.9235/0.9372	**0.9921/0.9909/0.9902**
Laplacian	0.9670**/**0.9680**/**0.9699	0.9260**/**0.9270**/**0.9301	0.8303/0.8449/0.8761	0.8611/0.8722/0.8977	0.9832/0.9833/0.9848

## Conclusion and Future Work

In the present study, we introduced a novel semi-supervised method called the semiMM that is based on spectral graph and mutual information theories and is used for gene selection. The semiMM method is a “filter” approach that simultaneously exploits local structure, variance, and mutual information. In the first step, we constructed a local nearest neighbour graph and subsequently divided this information into within-class and between-class local nearest neighbour graphs by weighing the edge between two data points. This method aims to discover the most discriminative features for classification by maximizing the local margin between within-class and between-class data, the variance of all data, and the mutual information of features with class labels.

In contrast to three state-of-the-art methods, i.e., the Fisher score, LS, and LSDF methods, the experimental results show that the semiMM method perfectly balances the use of both labelled and unlabelled samples. Regardless of whether the dataset is binary-class or multiclass, the proposed semiMM can always achieve a good performance. The performance of the semiMM is comparable to that of the Fisher score and even outperforms the Fisher score when the number of labelled samples equals 2, and the number of selected genes is less than 50. Both the Fisher score and semiMM are superior to the LS and LSDF in most cases.

The following issues should be addressed in future research:

No theoretical selection is established for the controlling parameter lambda, which tunes the weight between the first and second terms of the present criterion.

The semiMM considers only the discriminating information of class labels as features and between-labels. If this method can delete these redundant features, then a compact feature subset that is maximally discriminative and minimally redundant can be obtained.

The second term, which is the mutual information between class label and features, can be time-consuming when dealing with datasets with many subclasses. This factor makes the proposed semiMM method less competitive for multi-classification problems with limited time.

The analysis of single cell data has become a hot topic at present, and it is very interesting to extend the semiMM method to be used in the analysis of single cell data.
